# Accurate models vs. accurate estimates: A simulation study of Bayesian single-case experimental designs

**DOI:** 10.3758/s13428-020-01522-0

**Published:** 2021-02-11

**Authors:** Prathiba Natesan Batley, Larry Vernon Hedges

**Affiliations:** 1 Brunel University London, London, UK; 2 Department of Statistics, Northwestern University, Evanston, IL USA

**Keywords:** Single-case designs, Markov chain Monte Carlo (MCMC), Bayesian, Interrupted time-series models

## Abstract

Although statistical practices to evaluate intervention effects in single-case experimental design (SCEDs) have gained prominence in recent times, models are yet to incorporate and investigate all their analytic complexities. Most of these statistical models incorporate slopes and autocorrelations, both of which contribute to trend in the data. The question that arises is whether in SCED data that show trend, there is indeterminacy between estimating slope and autocorrelation, because both contribute to trend, and the data have a limited number of observations. Using Monte Carlo simulation, we compared the performance of four Bayesian change-point models: (a) intercepts only (IO), (b) slopes but no autocorrelations (SI), (c) autocorrelations but no slopes (NS), and (d) both autocorrelations and slopes (SA). Weakly informative priors were used to remain agnostic about the parameters. Coverage rates showed that for the SA model, either the slope effect size or the autocorrelation credible interval almost always erroneously contained 0, and the type II errors were prohibitively large. Considering the 0-coverage and coverage rates of slope effect size, intercept effect size, mean relative bias, and second-phase intercept relative bias, the SI model outperformed all other models. Therefore, it is recommended that researchers favor the SI model over the other three models. Research studies that develop slope effect sizes for SCEDs should consider the performance of the statistic by taking into account coverage and 0-coverage rates. These helped uncover patterns that were not realized in other simulation studies. We underline the need for investigating the use of informative priors in SCEDs.

Single-case experimental designs (SCEDs) involve manipulating an independent variable by applying an intervention to evaluate intervention effects by repeated, systematic measurements of an outcome variable (Horner et al., 2005; Kratochwill & Levin, 2014). Thus, SCEDs are forms of interrupted time-series designs, which are often used to evaluate intervention effects in various fields ranging across education (e.g. Lambert, Cartledge, Heward, & Lo, 2006), psychology (e.g. Shih, Chang, Wang, & Tseng, 2014), and medicine (as n-of-1 designs, Gabler, Duan, Vohra, & Kravitz, 2011). The importance and necessity of SCEDs in experimental designs where randomization is often impossible or inappropriate (e.g. low incidence disabilities, rare diseases, comorbid health conditions) has been discussed at length in SCED literature (e.g. Gast & Ledford, 2014; Kratochwill et al., 2010; Kratochwill & Levin, 2014; Shadish, 2014).

Often, visual analyses are conducted to analyze SCED data. These analyses are supplemented with reporting phase means, medians, percentages, and effect sizes such as standardized mean differences or indices based on the amount of data overlap between phases (Parker, Hagan-Burke, & Vannest, 2007). Although visual analysis has definite advantages with analyzing SCED data, studies have shown that the presence of autocorrelation can confound the results of visual analysis. For instance, in data with autocorrelation, it is difficult to decompose patterns due to trends (slopes) versus patterns due to autocorrelated errors. Autocorrelation is almost impossible to detect by visual analysis alone (Kazdin, 2011; Thyer & Myers, 2011). The presence of autocorrelation increases type I errors (Matyas & Greenwood, 1990) and decreases interrater reliabilities (Brossart, Parker, Olson, & Mahadevan, 2006) in visual analysis. In fact, Jones, Weinrott, and Vaughn (1978) found that in data with moderate-high autocorrelations, visual analysis results were reduced to nearly chance levels. Therefore, there is increasing emphasis for more objective methodologies for analyzing SCED data and determining causal inferences. Many organizations (American Speech-Language-Hearing Association, 2004; Cook et al., 2014; Kratochwill et al., 2013) have worked on reaching professional consensus on the methodological standards for SCEDs. One such standard, the U.S. Department of Education’s What Works Clearinghouse (WWC) pilot standards for single-case designs (Kratochwill et al., 2010) advocates that researchers evaluate the difference in levels, trends, and variability across phases in order to meet evidence standards for SCEDs. Therefore, it is somewhat common to see models with intercepts and slopes for each phase and the same autocorrelation for all phases being fitted to single-case experimental designs (Solomon, 2013). The multilevel model for SCEDs is an example of one such model (e.g. Baek & Ferron, 2013; Ferron, Bell, Hess, Rendina-Gobioff, & Hibbard, 2009; Ferron, Farmer, & Owens, 2010; van den Noortgate & Onghena, 2003a, b).

However, it may not be wise to fit such complex models to short time-series data. We hypothesize that this is because it is difficult to separate how much of the trend in the data is due to autocorrelation and how much is due to slope (i.e. a continuous gain or fall in the outcome variable). When fitting complex models to small sample data that are commonly found in SCEDs, we still do not know which parameters will be affected and to what extent they would be affected. The purpose of this simulation study is to investigate the performance of a two-phase interrupted time-series model with first-order autocorrelation in recovering the parameters of two-phase SCED data. We fit and compare four statistical models to SCED data with slopes and autocorrelations. The first model estimates slopes (commonly known as trends in SCED literature), intercepts (levels), and autocorrelations. The second model estimates only slopes and intercepts while assuming there is no autocorrelation. The third model estimates intercepts and autocorrelations assuming that any trend displayed by the data is due to autocorrelation and not slope. The fourth is the simplest model that estimates intercepts only and assumes that no trend is present, that is, there is no pattern due to autocorrelation or slope. We investigated which model best captures the data using diagnostics such as root mean squared errors (RMSEs) of the posterior means of slopes, intercepts, autocorrelations, and standard deviations; biases of slope and intercept effect sizes; coverage rates of the credible intervals (CI) of slopes, intercepts, and autocorrelations; and 0-coverage rates (that is, the percentage of CIs that contain 0) of slopes and autocorrelations. Bayesian estimation was used for all models because of its advantages with small sample data, especially SCEDs (e.g., Natesan Batley, 2020; Natesan Batley, Contractor, & Caldas, 2020; Natesan Batley, Minka, & Hedges, 2020; Natesan Batley, Shukla Mehta, & Hitchcock, 2020; Natesan, 2019; Natesan & Hedges, 2017; Rindskopf, 2014; Shadish, 2014; Shadish, Rindskopf, Hedges, & Sullivan, 2013). Readers are directed to the aforementioned references for further discussion of the role and advantages of Bayesian in estimating SCEDs.

## Literature review

In SCEDs, the intervention effect can manifest itself as change in level (Crosbie, 1995; Tryon, 1982) or change in trend (Crosbie, 1995; van den Noortgate & Onghena, 2003a, b) or a combination of both (Baek & Ferron, 2013). Thus, there are many ways of detecting intervention effects in SCEDs. These may utilize single-level or multilevel models. In the single-level model framework, Campbell and Stanley (1966) and Mood (1950) recommended testing whether the first observation of the intervention phase lay in the confidence interval of the predicted or extrapolated value at that time point assuming no intervention effect. If the true value of the first observation of the intervention phase lay in the confidence interval of the predicted value, a researcher may conclude that there was no intervention effect, whereas intervention effect may be tentatively inferred if otherwise. However, this procedure is weak because it does not make use of all data points (Campbell & Stanley, 1966). Therefore, another option is to compare the intercepts and slopes of the regression lines of both phases. If the intercepts and slopes are the same, the null hypothesis that the treatment is not effective cannot be rejected (Campbell, 1967). Algina and Swaminathan (1977) showed that the test statistic for testing the intervention effect in single-group quasi-experimental time-series designs for linear trends follows the F-distribution. However, this is confounded by autocorrelation because the measurements are obtained on an individual across time. Ignoring autocorrelations will lead to biased parameter and standard error estimates, which in turn, hinders the validity of statistical inferences (Pankratz, 1983).

All the aforementioned procedures ignore autocorrelation. Trend in data with autocorrelations leads to under- or overestimated treatment effect sizes (West & Hepworth, 1991). The presence of autocorrelations biases error variances, confidence intervals, *t* values, and type I error rates (Glass, Willson, & Gottman, 1975; Gottman, 1980; Gottman & Glass, 1978; McCain & McCleary, 1979). Gottman and Glass (1978) showed that the type I error of a *t* test with alpha level = 0.05, when the autocorrelation is 0.5, is 0.2584. Similarly, Hibbs (1974) concluded that the type I error rate is inflated by 265% when the autocorrelation is 0.7. Huitema, McKean, and McKnight (1999) showed that ordinary least squares estimates of slopes have higher type I errors for larger values of positive autocorrelation, especially for large sample sizes. This is because the variance of the slope is underestimated in the presence of positive autocorrelations. This in turn affects the slope change parameters whose error rates were unacceptably high for autocorrelations greater than 0.20. Finally, positive autocorrelations were associated with higher type I error rates for estimates of slope change than for estimates of level change. Therefore, Huitema, McKean, and McKnight (1999) concluded that large sample theory overestimates the harmful effects of autocorrelation of type I error in small samples.

Glass, Willson, and Gottman (1972) adopted autoregressive (AR) and autoregressive integrated moving average (ARIMA) processes for testing intervention effects in time-series data. Simonton (1977) outlined a procedure for comparing the regression lines of an interrupted time-series model assuming first-order autocorrelations. However, this procedure requires the number of individuals to be greater than the number of measurement occasions. Obviously, this requirement is almost impossible to fulfill in single-case experimental designs. Other researchers have suggested that a minimum of 50 observations is required to obtain sufficiently accurate estimates for a first-order autoregressive model (Box & Pierce, 1970; Glass, Willson, & Gottman, 1975; Ljung & Box, 1978).

Huitema and McKean (2000) studied the two-phase interrupted time-series model and recommended that individual slopes and intercepts be estimated for each phase. However, this study was conducted in the absence of autocorrelation. McKnight, McKean, and Huitema’s (2000) double bootstrap method had a bias in autocorrelation estimate ranging from 0.018 to 0.2 for a time-series length of 20. The bias decreased with increase in time-series length. However, SCED data are often even shorter in length. In fact, in a systematic literature review by Shadish and Sullivan (2013) of the SCED articles published in 2008, excluding alternating treatments design, only 54.7% of the 563 articles had more than five data points in the baseline phase. The median number of total data points in 809 studies was 20, and 90.6% had fewer than 50 data points in total. This leaves fewer than 25 data points per phase if the designs only had two phases, and even fewer data points in designs with more than two phases. Approximately 70% of the studies had fewer than 30 data points in all.

There has been considerable effort in developing methods and effect sizes for SCED data with trend (e.g. Allison & Gorman, 1993; Center, Skiba, and Casey, 1985-86; Gorman & Allison, 1997; White, Rusch, Kazdin, & Hartmann, 1989). Researchers van den Noortgate and Onghena (2003a) discussed procedures for meta-analyses with linear trends. Parker, Vannest, and Davis (2011) developed a method to control positive baseline trend within data non-overlap. However, this procedure ignores the presence of autocorrelation and capitalizes on overlap indices, which are known to have many drawbacks in addition to ignoring the distances between data points and being sensitive to outliers. Parker, Vannest, Davis, and Sauber (2011) developed an effect size that combined trend and autocorrelation in SCED data. Cobb and Shadish (2015) and Sullivan, Shadish, and Steiner (2015) used semi-parametric regression models to analyze SCED data with linear and nonlinear trends. Beretvas and Chung (2008) used the difference in *R*^2^ (Δ*R*^2^) as an index of effect size, which is an indicator of change in both intercepts and slopes for single-case designs with trends. Their results showed that Δ*R*^2^ has acceptable statistical properties only in the absence of autocorrelation and has poor performance in the presence of autocorrelation, especially for few cases and few time points. Specifically, for large values of autocorrelations, the type I error rate, that is, rejecting the null hypothesis that there is no intervention effect based on Δ*R*^2^, was high. Whether this is because the autocorrelations ended up being estimated as slopes is unknown. Solanas, Manolov, and Onghena’s (2010) and Manolov and Solanas’ (2009) model with slope and autocorrelation eliminates baseline trend from SCED data to estimate slope and level changes. However, they diagnosed the performance of the models only using biases, which do not shed light on whether the trend in the data was appropriately decomposed into autocorrelations and slopes. Without examining the interval estimates of slopes and autocorrelations, it is impossible to tell if there is an indeterminacy problem, that is, whether sometimes slope is estimated as autocorrelation and vice versa. This is important because autocorrelation is not considered as part of the intervention effect, whereas change in slopes is usually attributed to the intervention effect in SCEDs. In sum, although parametric approaches based on regression have great promise for meta-analysis of SCEDs, we are yet to know the full extent of their weaknesses and strengths.

Although the performance of models that estimate trends and autocorrelations have been investigated using the multilevel modeling (MLM) framework, the present study is a development over these studies because they had some limitations. For instance, Ferron, Farmer, and Owens (2010) studied MLM for multiple-baseline designs and compared different approaches to show that estimates and coverage rates improved with phase length and effect size. Similarly, Ferron, Bell, Hess, Rendina-Gobioff, and Hibbard (2009) showed that although the treatment effect estimates were relatively accurate in the presence of autocorrelation, the point estimates were biased. However, the aforementioned studies (i.e. Ferron, Bell, Hess, Rendina-Gobioff, & Hibbard, 2009; Ferron, Farmer, & Owens, 2010) are applicable only to multiple-baseline designs and required at least eight participants for robust estimation of parameters. Moeyaert et al. (2015) demonstrated multilevel meta-analysis of results from various types of SCEDs. Petit-Bois, Bark, Van den Noortgate, Beretvas, and Ferron (2016) conducted a simulation of meta-analysis of 10 or 30 studies and used sample sizes of four and seven. Thus, they had much larger data. Although Ugille, Moeyaert, Beretvas, Ferron, and Van den Noortgate (2012) showed that MLM can be applied to datasets with as few as four participants, and a time-series length of 10 series per SCED study, this still places greater burden on the researcher in terms of data collection. This is because a minimum of three participants and five data points per phase are required to meet the WWC design standards. Fewer than 63% of the studies reviewed by Shadish and Sullivan (2013) had 20 or more data points in total. What the present study solves is a much more basic problem when considering any multiphase design for one participant using the simpler, single-level model where MLM is not applicable. The advantage of our approach is that the findings from our study are applicable to a wider set of SCEDs such as the ABAB design the changing criterion design, the multiple-baseline design, or the alternating treatments design. Moreover, although some of the abovementioned studies examined the coverage rates of autocorrelations, none of them examined 0-coverage of autocorrelation. Examining 0-coverage is important because it tells us if the estimated value of autocorrelation is incorrectly computed as 0 (i.e. being nonexistent). On the contrary, coverage rates only tell us if the true value is contained in the interval estimate.

In data that exhibit both slopes and autocorrelations, a model that neglects slope is expected to produce strongly autocorrelated residuals (Shadish, Rindskopf, & Hedges, 2008). This may be because the pattern in the data due to the slopes is estimated as the pattern in the data due to autocorrelation. Thus, Simonton (1979) questioned the specific advantages that accrue from augmented complexity in short time-series data. Huitema, McKean, and McKnight (1999) also seconded this opinion and asked whether complex approaches are necessary when modeling the dependency structure of observations in time-series designs. Specifically, the question remains as to whether it is prudent to fit models with slopes and intercepts that vary by phase and an autocorrelation that is common to all phases for SCED data, which are short time-series data, let alone develop effect sizes, and multi-level models. Simpler models generally have greater statistical power and are simpler to interpret. However, the sensitivity of these models to violation of assumptions such as independence of observations needs to be studied further before they can be recommended for general use. This forms the impetus for the present study.

## Models

A continuous, normally distributed dependent variable with slope and autocorrelation was considered as the outcome variable in the present study. Four single-level Bayesian models were fitted to the data as shown in Table 1. These models varied based on whether slopes and autocorrelations were estimated in the model or not.

**Table 1:** Bayesian models fitted in the study

		Slope	
		No	Yes
Autocorrelation	No	IO (intercepts only)	SI (slopes and intercepts only)
	Yes	NS (no slopes)	SA (slopes and autocorrelations)

### Model 1 (Intercepts, slopes, and autocorrelations – SA model)

The SA model estimates intercepts, slopes, and autocorrelations. Consider a SCED with two phases: baseline and treatment. Let the time points in the baseline phase be 1, 2, …, *t*_*b*_ and in the treatment phase be *t*_*b* + 1_, …, *t*_*n*_. Let us assume that the observed value at the first time point (*y*_*p*1_) in phase *p* follows a normal distribution with the mean of ${\hat{y}}_{p1}$ and standard deviation of *σ*_*ε*_ as shown in Eq. 1.

1 $$ {y}_{p1}\sim \mathit{\operatorname{norm}}\left({\hat{y}}_{p1},{\sigma}_{\varepsilon}^2\right). $$

The predicted values in the following time points *t* are distributed as:

2 $$ {y}_{pt}\mid {H}_{pt-1},\Theta \sim \mathit{\operatorname{norm}}\left({\hat{y}}_{pt\mid \left( pt-1\right)},{\sigma}_e^2\right). $$

In Eq. 2, *H*_*pt* − 1_ is the past history, Θ is the vector of parameters, and *σ*_*e*_ is the white noise created by a combination of random error (${\sigma}_{\varepsilon}^2$) and autocorrelation between adjacent time points, *ρ*. The SA model and the serial dependency of the residual (*e*_*t*_) can be expressed as:

3 $$ {\hat{y}}_{pt}=\left\{\begin{array}{c}{\beta}_{11}+{\beta}_{21}t+\varepsilon +\rho {e}_{pt-1}, if\ t\le {t}_b\\ {}{\beta}_{12}+{\beta}_{22}\left(t-{t}_b\right)+\varepsilon +\rho {e}_{pt-1}, otherwise\end{array}\mathrm{and}\right. $$

4 $$ {e}_{pt-1}=\rho {e}_{pt-2}+\varepsilon . $$

In Eq. 3, ${\hat{y}}_{pt}$ is the probability of the predicted value of the dependent variable at time *t* in phase *p*; *β*_11_ and *β*_21_ are the intercept and slope of the linear regression model for phase 1, respectively; *β*_12_ and *β*_22_ are the intercept and slope of the linear regression model for phase 2, respectively; *e*_*pt*_ is the error at time *t* in phase *p*; *ρ* is the autocorrelation coefficient which stays the same across both phases; and *ε* is the independently distributed error. The standard deviations of *e*, *ε*, *and ρ* are related as shown in Eq. 5.

5 $$ {\sigma}_e=\frac{\sigma_{\varepsilon }}{\sqrt{1-{\rho}^2}}. $$

The intercept and slope *β*_1*p*_ and *β*_2*p*_ can be modeled as:

6 $$ {\beta}_{ip}=\left\{\begin{array}{c}{\beta}_{i1}, if\ t\le {t}_b\\ {}{\beta}_{i2}, otherwise\end{array}\right., $$

where the terms refer to intercepts when *i* = 1 and slopes when *i* = 2. Intercept effect size *ES*_1_ was defined as the standardized mean difference between the two phases as given in Eq. 7. Slope effect size *ES*_2_ was defined as the difference between the estimated value at the midpoint of the intervention phase assuming and not assuming an intervention effect as shown in Eq. 8. If *t*_*i*_ is the number of time points in the intervention phase, then

7 $$ {ES}_1=\frac{\beta_{12}-{\beta}_{11}}{\sigma_{\varepsilon }}\ \mathrm{and} $$

8 $$ {ES}_2=\left({\beta}_{12}+\left({t}_b+\frac{t_i}{2}\right){\beta}_{22}\right)-\left({\beta}_{11}+\left({t}_b+\frac{t_i}{2}\right){\beta}_{21}\right). $$

### Model 2 (Intercepts and autocorrelations but no slopes – NS model)

The NS model assumes that any trend in the data is due to autocorrelation and not a slope parameter. Thus, the *β*_21_ and *β*_22_terms are dropped or equal 0 in Eqs. 3 and 6, and only intercepts and autocorrelations are estimated. Thus, Eq. 3 becomes

9 $$ {\hat{y}}_{pt}=\left\{\begin{array}{c}{\beta}_{11}+\varepsilon +\rho {e}_{pt-1}, if\ t\le {t}_b\\ {}{\beta}_{12}+\varepsilon +\rho {e}_{pt-1}, otherwise\end{array}\right.\ \mathrm{and} $$

### Model 3 (Slopes and intercepts but no autocorrelation – SI model)

The slopes model assumes that the data are not autocorrelated. Thus, the *ρ* term vanishes or equals 0, thereby making Eqs. 1–6 represent a simple piecewise regression model where only slopes and intercepts are estimated. The model becomes

10 $$ {\hat{y}}_{pt}=\left\{\begin{array}{c}{\beta}_{11}+{\beta}_{21}t+\varepsilon, if\ t\le {t}_b\\ {}{\beta}_{12}+{\beta}_{22}\left(t-{t}_b\right)+\varepsilon, otherwise\end{array}\right.. $$

### Model 4 (Intercepts only and no autocorrelations or slopes – IO model)

The IO model is the simplest of all models where no trend is assumed. Therefore, both slopes and autocorrelations are set to 0, and only intercepts are estimated. Thus, the variability in the data is only due to random error as shown in Eq. 11.

11 $$ {\hat{y}}_{pt}=\left\{\begin{array}{c}{\beta}_{11}+\varepsilon, if\ t\le {t}_b\\ {}{\beta}_{12}+\varepsilon, otherwise\end{array}\ \mathrm{and}\right. $$

### Priors

The priors were the same for the parameters that were common to all the models. We used weakly informative priors, which purposely include less information than what we actually have (Gelman & Jakulin, 2007). This allows the parameters of the priors to be estimated from the data rather than specifying them to have subjective information, especially for small sample data like those in the present study (Efron & Morris, 1975; Gelman, 2006; James & Stein, 1960). The intercepts and slopes of both phases were independent of each other. The intercepts and slopes are drawn from normal distributions with hyperpriors in order to reduce the impact of the prior specification on the estimates as given in Eqs. 12–15. The variances of the intercepts and slopes were independently drawn from gamma distributions with mode and standard deviations ranging uniformly between 0.01 and 1.^1^ We chose the means of the intercepts to come from a distribution that uniformly ranged from 0 to 50 because we assume that the mean of the dependent variable would not be outside these bounds based on the simulation design. Of course, practitioners should choose appropriate priors for their data depending on the scale of the outcome variable. For instance, the mean of an outcome variable such as the number of problem behaviors exhibited by a child with autism during an observation period might range from 0 to an upper limit that makes substantive sense. The means of the slope parameters were sampled from a unit normal distribution because this value indicated change in the outcome variable which might be positive or negative and included all plausible values for means of the slopes based on the simulation parameters.

12 $$ {\beta}_{1p}\sim \mathit{\operatorname{norm}}\left({\mu}_{1p},{\sigma}_{int}^2\right) $$

13 $$ {\beta}_{2p}\sim \mathit{\operatorname{norm}}\left({\mu}_{2p},{\sigma}_{slope}^2\right) $$

14 $$ {\mu}_{1p}\sim unif\left(0,50\right);p=1,2 $$

15 $$ {\mu}_{2p}\sim \mathit{\operatorname{norm}}\left(0,1\right);p=1,2 $$

Other prior specifications were as follows:

16 $$ {\sigma}_{\varepsilon}\sim unif\left(0.1,5\right)\ \mathrm{and} $$

17 $$ \rho \sim unif\left(-1,1\right). $$

## Method

Data were simulated for the following conditions for an interrupted time series model. Phase length (*l*): 5, 8, 10, 15; standard deviation (*σ*_*ε*_): 1, 2, 5; intercept effect size (*ES*_1_): 0.5, 1, 2, 5; slope effect size (*ES*_2_): 0, 0.3, 0.5, 1; and autocorrelations (*ρ*): 0, 0.2, 0.5, 0.8. Therefore, this was a fully crossed 3 × 4 × 4 × 4 × 4, resulting in 768 conditions. One hundred datasets were generated for each condition, yielding 76,800 datasets. Some of the data conditions such as phase length, standard deviation, intercept effect size, and autocorrelations were chosen based on previous literature (Natesan & Hedges, 2017; Natesan Batley, Minka, et al., 2020). The four models discussed in the models section were each fitted to each dataset. Root mean squared errors (RMSEs), mean relative biases, and coverage rates of the intercepts, slopes, intercept and slope effect sizes, autocorrelations, and standard deviations were used to compare the performance of the models. RMSEs are defined as the square root of the average squared deviation of the estimated value from the true value over all replications for a given data condition. Mean relative bias is computed as the average of the ratio of the difference between the true and the estimated value of a parameter. RMSE and relative bias for a parameter *x* whose estimate in the *i*th replication is *x*_*i*_ and true value is *X* over *n* replications is given in Eqs. 18 and 19.

18 $$ RMSE=\sqrt{\frac{\sum_{i=1}^n{\left({x}_i-X\right)}^2}{n}}\mathrm{and} $$

19 $$ Relative\ bias=\frac{1}{n}\frac{\sum_{i=1}^n\left({x}_i-X\right)}{X}. $$

Larger RMSEs and larger mean relative biases indicate less accurate estimates. According to Hoogland and Boomsma (1998), any relative bias greater than 0.05 was substantial in covariance structure models. We note here that relative bias was not computed for conditions where the true value was 0. Coverage rates are defined as the percentage of interval estimates that contain the true parameter value. 0-Coverage rates, that is, the percentage of credible intervals (CI) that contained 0 were used to examine if there was indeterminacy between estimating the slope and the autocorrelation. That is, when the credible interval of the autocorrelation contains 0 when it is not expected to, the trend in the data may be incorrectly or inaccurately attributed to slope and the vice versa. This is represented as 0-coverage and represented as the parameter estimate followed by “−0” (e.g. *ρ* − 0). However, if both slope and autocorrelation credible intervals contain 0, this signals that in some iterations, autocorrelation takes more credit for the trend in the data (while the slope is estimated to be 0), and in some iterations slope takes more credit for trend in the data (while the autocorrelation is estimated to be 0).

### Adequacy of iterations and replications

**Fig. 1:**
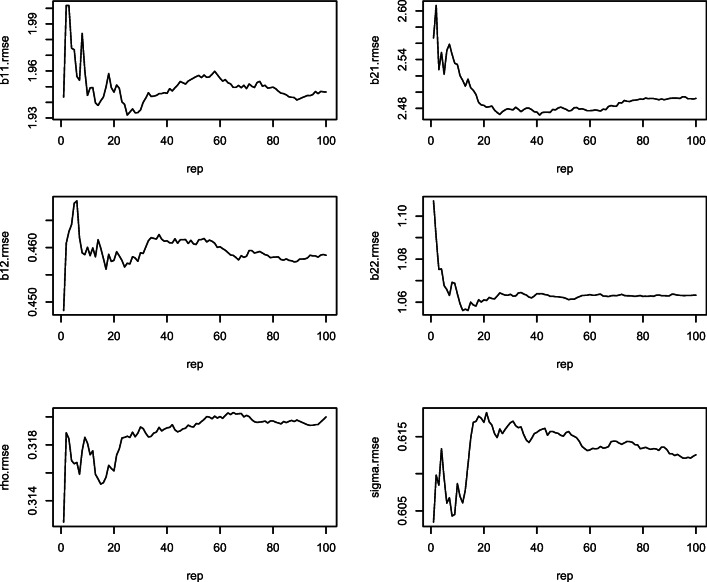
RMSEs versus replications for all parameter estimates for the SA model. X-axis represents the replication dataset, and the Y-axis represents the RMSEs of various parameters

The R computing environment was used for simulation and data analysis (R, 2014). The package RunJAGS (Denwood, 2013), conveniently called JAGS (Plummer, 2003), runs parallel chains and iterates the model estimates until convergence is indicated. Four parallel chains were run with starting values independently generated for each chain from the prior distribution. The first 100,000 iterations were discarded using the burn-in procedure. Convergence was checked using two convergence diagnostics: the multivariate potential scale reduction factor (MPSRF, Brooks & Gelman, 1998), and Heidelberger and Welch’s (1983) convergence diagnostic. In order to determine the adequacy of 100 replications (datasets) per condition, RMSEs and coverage rates of intercepts, slopes, intercept and slope effect sizes, autocorrelations, and standard deviations of the most complex model (SA) were plotted against the number of datasets generated. This procedure is similar to the one proposed by Koehler, Brown, and Haneuse (2009). When the RMSEs and coverage rates stopped fluctuating wildly or appeared to converge, there was indication of sufficient number of replications. This indicated that running the simulation for more datasets would not contribute to better diagnostic estimates such as RMSEs. In our study, 100 replications per data condition were deemed sufficient. The cumulative RMSEs and the coverage rates appeared to stop fluctuating significantly after the first 40 replications for all parameters. The cumulative RMSEs of all parameters fluctuated less than 0.03 after the first 60 iterations, as shown in Fig. 1. The pattern for coverage rates was similar. We also stopped at 100 replications because of the computationally intensive nature of the estimation. It took 45 days to estimate all parameters of the four models across all 76,800 datasets on six computers, each with quad core processors. We used doParallel and foreach (Weston & Calway, 2017) to parallelize the replications across the processors. Independent ANOVAs were conducted to measure the effect of the various data conditions on the RMSEs and coverage rates of the parameters. The data conditions were the independent variables. Eta-squared was computed for all main and two-way interaction effects. Plots were examined to understand the patterns in parameter recovery.

## Results

### Overall trends from the ANOVAs

The eta-squared effect sizes from independent ANOVAs are given in Tables 2 and 3. These values give us a general pattern of those data conditions that affected the RMSEs, mean relative biases, coverage rates, means, and mean posterior standard deviations.

**Table 2:** Eta-squared effect sizes of ANOVAs of RMSEs and mean biases in %

	Diagnostic statistic	RMSE						Mean bias		Relative bias	
	Parameter	*ρ*	*β*_11_	*β*_21_	*β*_12_	*β*_22_	*σ*_*ε*_	*ES*_2_	*ES*_1_	*ES*_2_	*β*_21_
	Models compared	NS vs. SA	All	All	SI vs. SA	SI vs. SA	All	SI vs. SA	All	SI vs. SA	All
Effects	Length	34.72			24.51	21.89					
	sigma		62.77	53.00	50.87	48.44		39.82		29.41	4.02
	Int.es			17.78			46.05	6.82	88.74	6.54	29.56
	Slope.es									4.91	
	rho	7.06	18.80		10.36	5.66					
	Model						18.13				30.09
	Length × rho	16.30									
	sigma × int.es			9.92				11.58		9.97	
	Int.es × model			5.24			21.80		4.56		
	rho × model	8.56									
	rho × sigma		7.37								
	Length × sigma					6.61					

Note: Only effect sizes larger than 4% are shown. NS is the no slopes but autocorrelations model. SA is the slopes and autocorrelations model. *β*_11_ and *β*_21_ are the intercepts of the first and second phases, respectively. *β*_12_ and *β*_22_ are the slopes of the first and second phases, respectively

**Table 3:** Eta-squared effect sizes of ANOVAs of coverages and mean standard deviations (SDs) in %

	Diagnostic statistic	Coverage								Mean SD	
	Parameter	*ρ* − 0	*ρ*	*ES*_2_ − 0	*ES*_2_	*β*_11_	*β*_21_	*β*_12_ − 0	*β*_22_ − 0	*β*_11_	*β*_21_
	Models compared	NS vs. SA	NS vs. SA	SI vs. SA	SI vs. SA	All	All	Only SA	Only SA	All	All
Effects	Length	28.81						8.00	7.38		
	Sigma									69.26	65.92
	Int.es						22.59		59.95		
	Slope.es										
	Rho	37.55	20.22	33.79	34.77	34.63		25.11			
	Model	5.94	62.03	28.54	27.36	26.97	38.52			16.43	15.95
	Length × rho	14.47									
	Sigma × int.es										
	Int.es × model						13.76				
	Rho × model		15.67	19.42	19.7	22.33	5.14				
	Length × int.es								7.87		
	Rho × int.es								4.02		

Note: Only effect sizes larger than 4% are shown. NS is the no slopes but autocorrelations model. SA is the slopes and autocorrelations model. SI is the slopes and intercepts model. *ES*_2_ − 0 represents the 0-coverage rates of slope effect size. *β*_11_ and *β*_21_ are the intercepts of the first and second phases, respectively. *β*_12_ and *β*_22_ are the slopes of the first and second phases, respectively

#### Autocorrelations

**Fig. 2:**
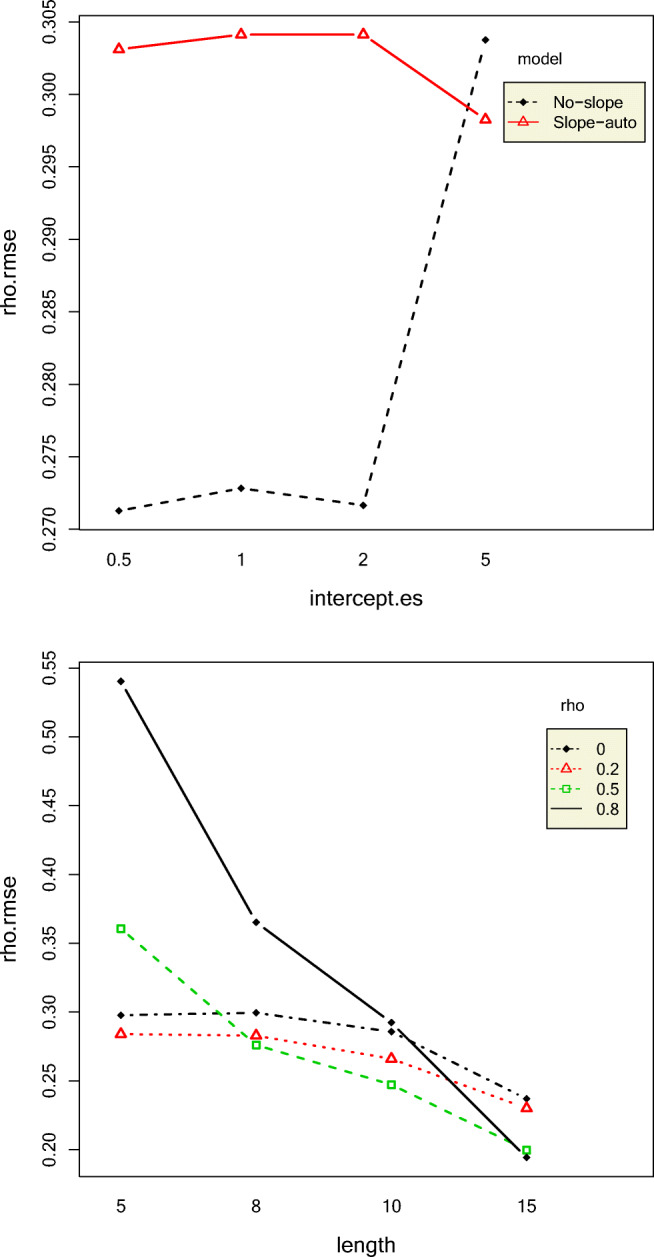
Top panel: Two-way interaction effects of intercept effect size and model on rho RMSE Bottom panel: Two-way interaction effects of phase length and autocorrelation on rho RMSE

**Fig. 3:**
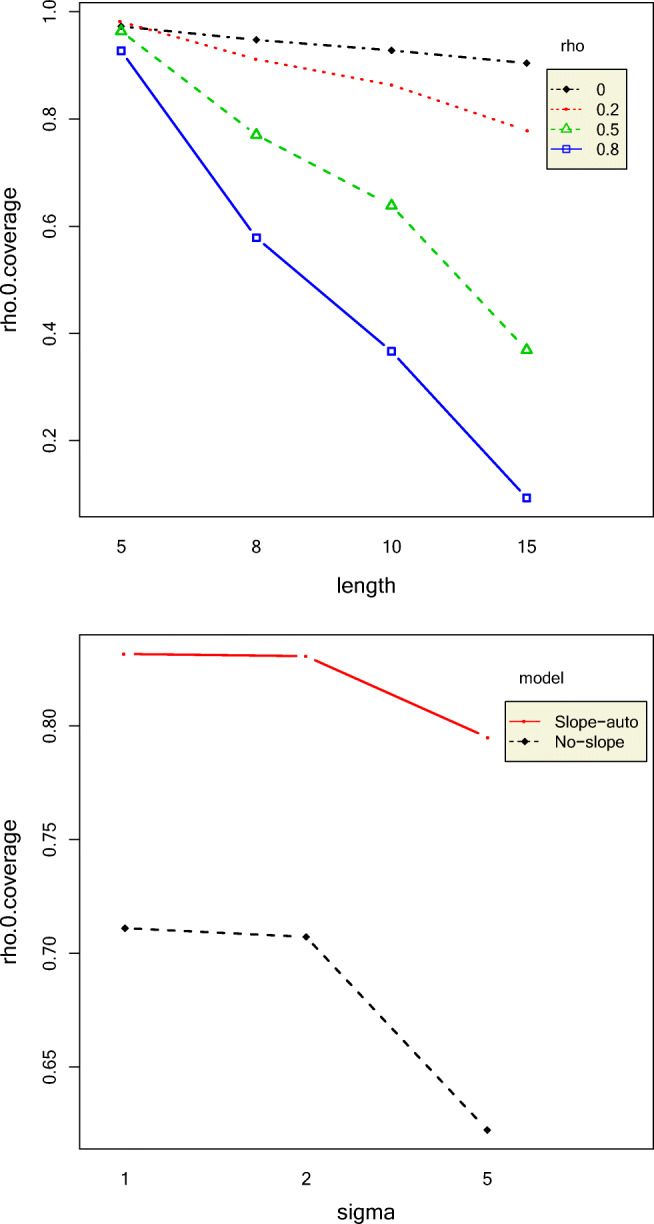
Top panel: Two-way interaction effects of phase length and autocorrelation on rho 0-coverage. Bottom panel: Two-way interaction effects of standard deviation and model on rho 0-coverage rate

**Fig. 4:**
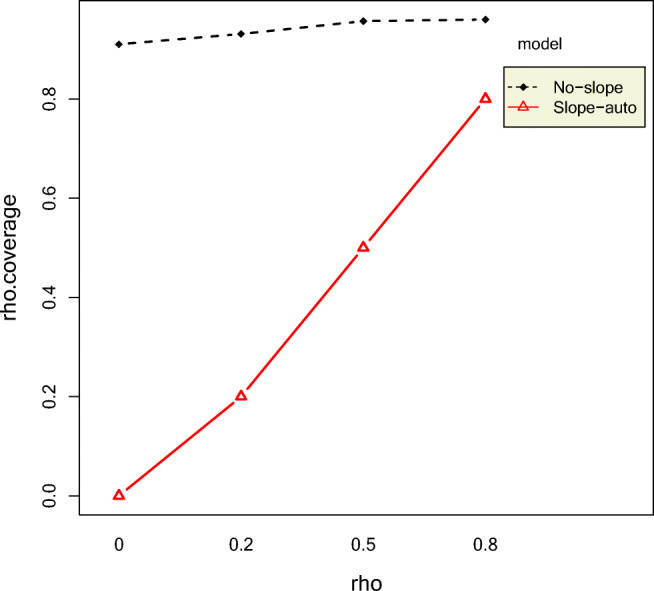
Two-way interaction effects of autocorrelation and model on rho coverage rate

**Fig. 5:**
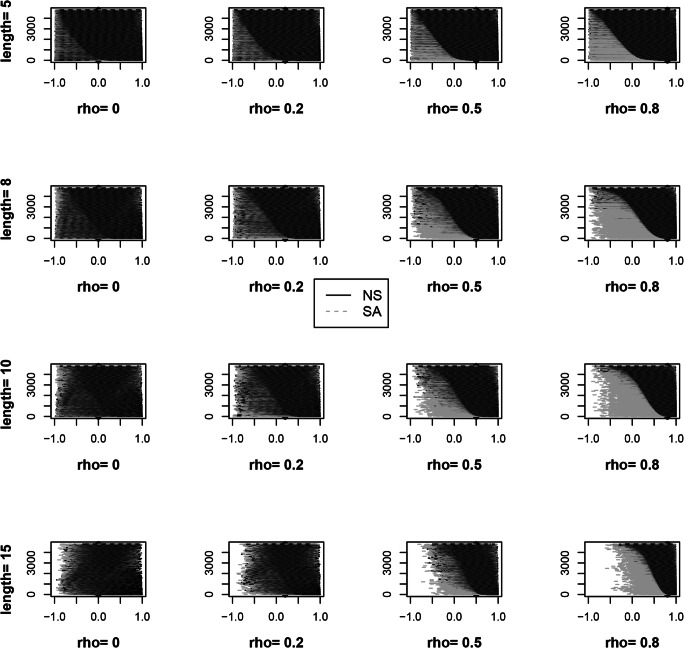
Credibility intervals of NS and SA autocorrelation estimates showing shorter CIs for NS. Y-axes represent repetitions. Each horizontal line represents the credibility interval obtained from a dataset. The intervals are ordered by widths

Longer phase lengths yielded smaller RMSE autocorrelations, indicating that longer time-series yield more accurate estimates. However, phase lengths did not affect the coverage rates of autocorrelations with the exception of high 0-coverage rates for longer phase lengths combined with high autocorrelations. The *ρ* RMSEs were always larger for the NS model compared to the SA model, especially for larger values of intercept effect size. The interaction effect between autocorrelation and model accounted for 8.56% of the variation in *ρ* RMSE. This is shown in Fig. 2. Estimates from datasets with longer phase lengths combined with larger autocorrelation values covered 0 less frequently than those with smaller phase lengths and smaller autocorrelation values, as shown in Fig. 3. The NS model had lower 0-coverage rates but substantially higher coverage rates of autocorrelation than the SA model, as shown in Fig. 4. Even for an original autocorrelation value of 0.8, 60% of the SA model's CIs contained 0. Coverage rates of autocorrelation increased with increase in true *ρ* value for both NS and SA models, but the increase in coverage rate was more rapid for the SA model. The NS model had narrower CIs than the SA model, as shown in Fig. 5. The width of the CIs increased with increase in phase length and decrease in autocorrelation.

#### Slopes

**Fig. 6:**
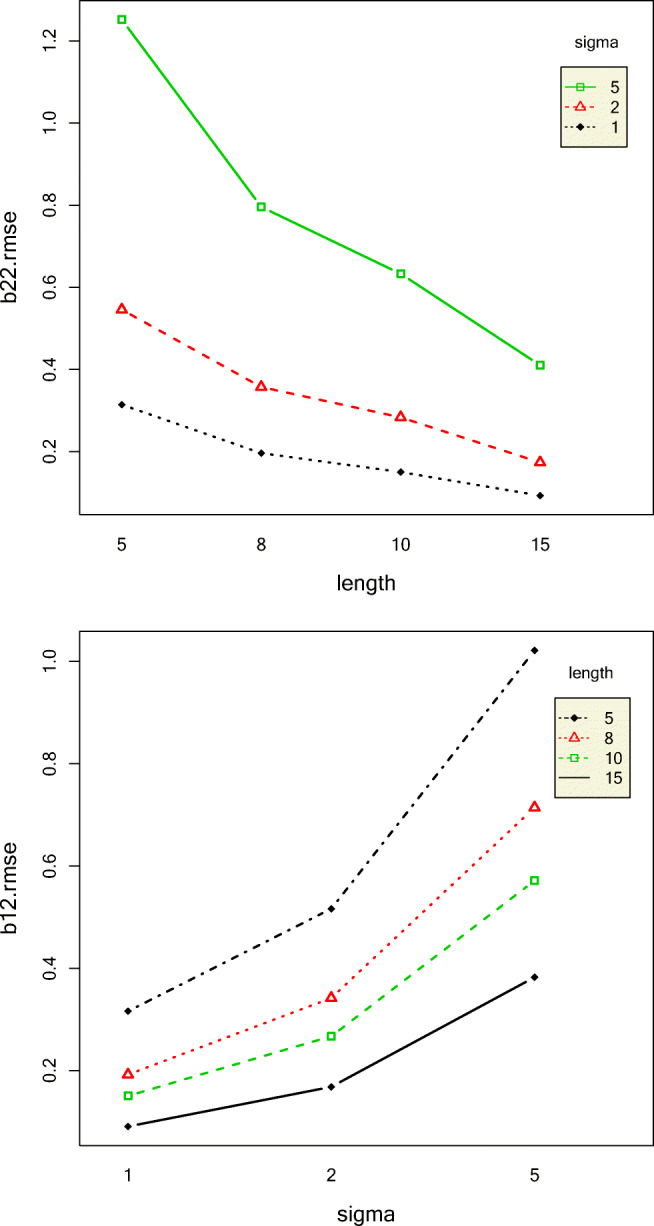
Two-way interaction effects of length and sigma on the RMSEs of the slopes of the first phase (top) and the second phase (bottom)

SI and SA models were compared for their recovery of slopes and slope effect sizes. RMSE of the first phase slopes (*β*_12_) decreased with increase in phase length and decrease in standard deviation and autocorrelation, as shown in Fig. 6. This makes intuitive sense because longer time-series, smaller standard deviations, and lower autocorrelations all contribute to clearer patterns, and hence, smaller slope RMSEs. The SA model had slightly lower *β*_12_ RMSE than the SI model, but this effect was very small. The RMSE of the second-phase slope *β*_22_ was impacted most by variation in standard deviation and phase length. *β*_22_ RMSE decreased with increase in phase length and decrease in standard deviation.

#### Intercepts

**Fig. 7:**
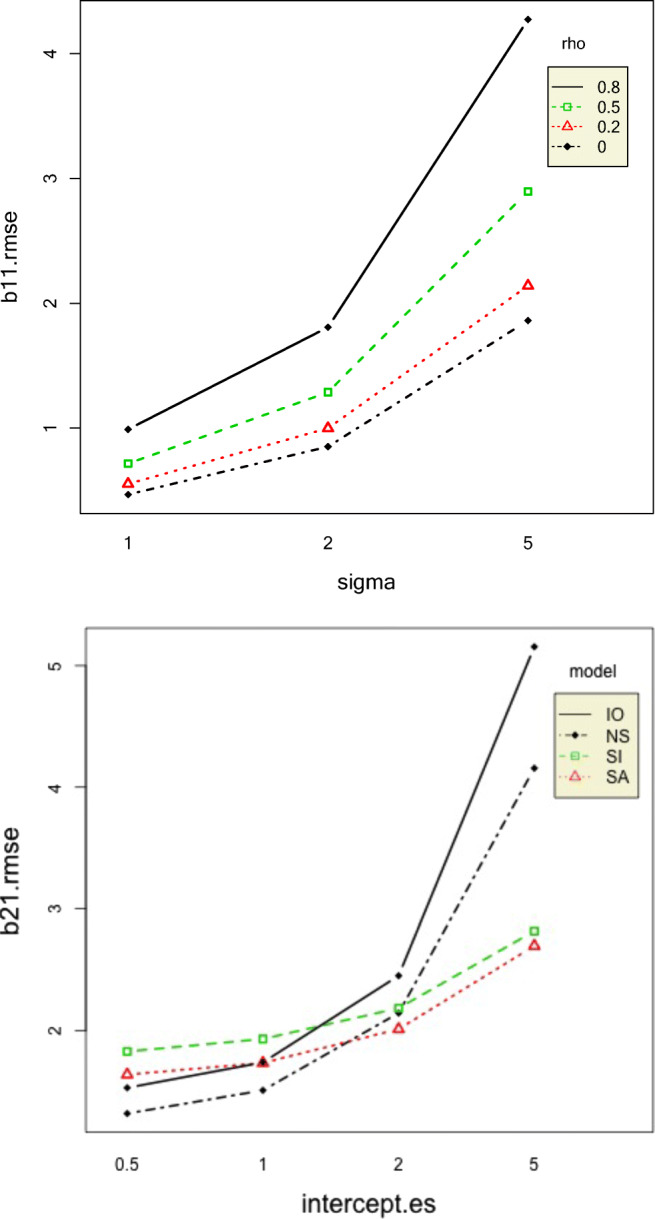
Top panel: Two-way interaction effect of sigma and autocorrelation on the RMSE of the first-phase intercept. Bottom panel: Two-way interaction effect of intercept effect size and model on the RMSE of the second-phase intercept

**Fig. 8:**
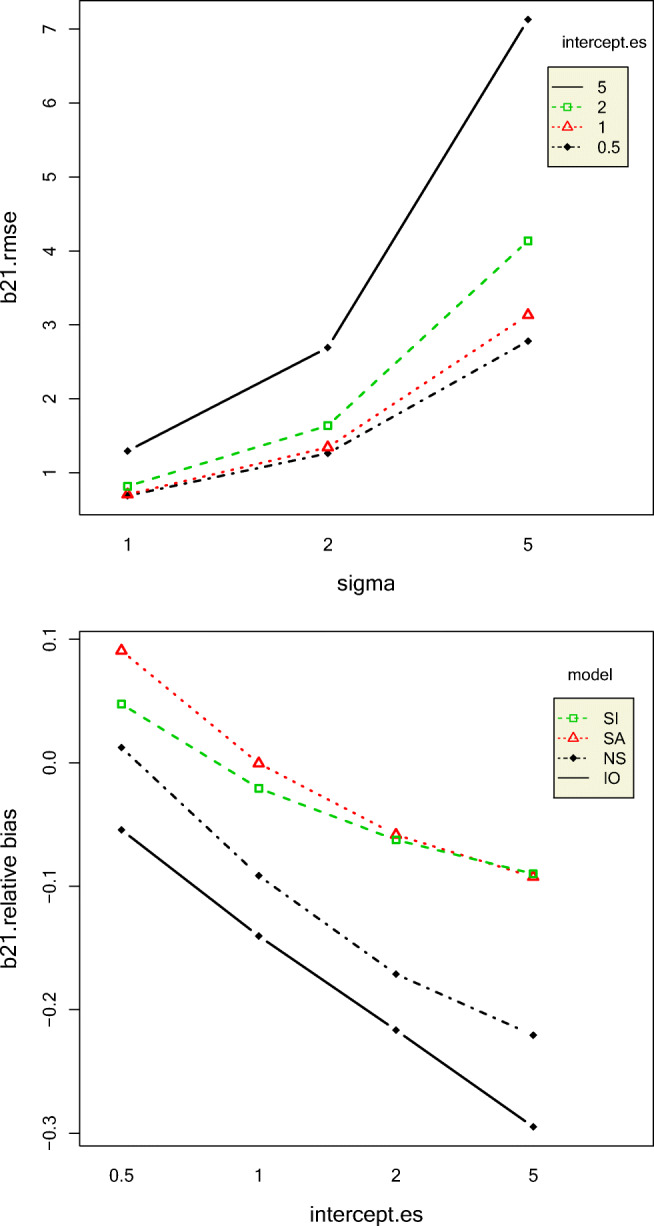
Top panel: Two-way interaction effect of sigma and intercept effect size on the RMSE of the second-phase intercept. Bottom panel: Two-way interaction effect of intercept effect size and model on the relative bias of the second-phase intercept

Standard deviation, autocorrelation, and their interaction explained most of the variation in the RMSE of the intercept of the first phase *β*_11_. The RMSEs of *β*_11_ increased with increase in standard deviation and autocorrelation which is to be expected because increase in both these data conditions leads to less clear data patterns. The RMSEs of the second-phase intercept *β*_21_ increased with increase in standard deviation and intercept effect size. The interaction effects between intercept effect size and standard deviation and intercept effect size and model also had a substantial effect on the RMSE of *β*_21_. For intercept effect sizes up to 2, *β*_21_ RMSE was similar for all models but rapidly increased for the IO model followed by NS, SI, and SA models, as shown in Fig. 7. It might seem illogical that with increase in intercept effect size, the *β*_21_ RMSE would increase because larger intercept effect size would indicate a clearer pattern. To understand this result more, we computed the mean relative bias of *β*_21_. The mean relative bias of *β*_21_increased with increase in intercept effect size, as shown in Fig. 8. However, the absolute value of mean relative biases were less than 0.05 only for small values of intercept effect size for only SI and NS models.

#### Slope and intercept effect sizes

**Fig. 9:**
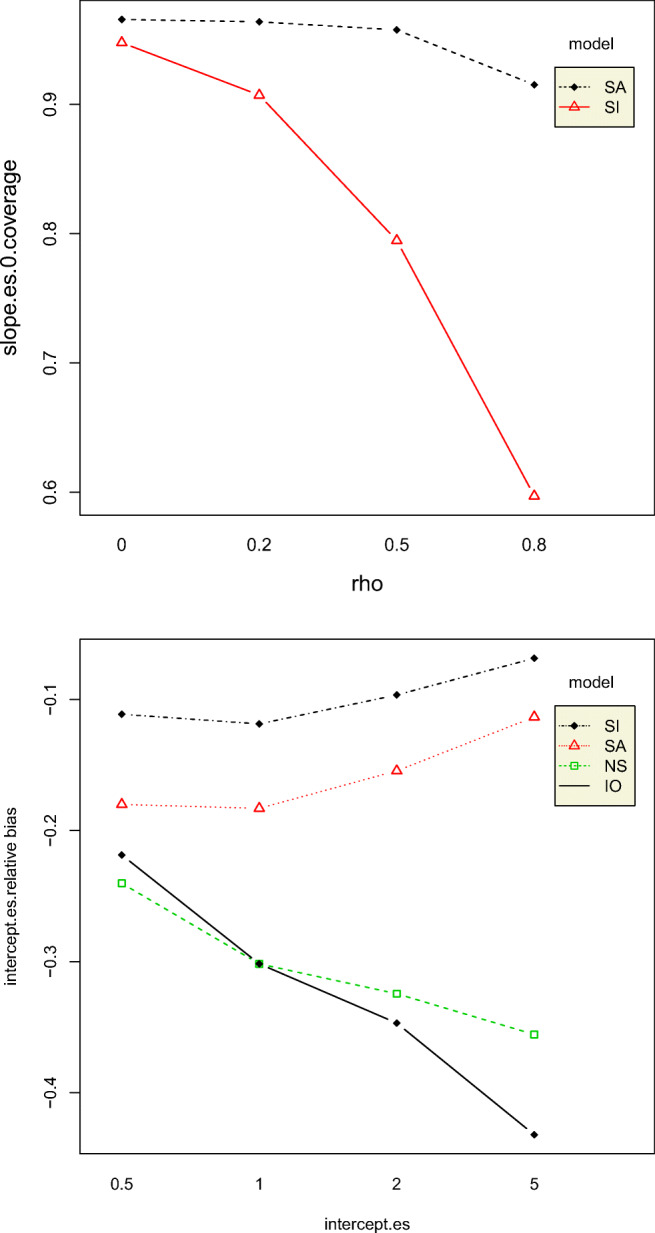
Two-way interaction effects of intercept effect size and model on intercept effect size relative bias (left) and autocorrelation and model on the slope effect size 0-coverage

The mean bias of the slope effect size (*ES*_2_), that is, the mean difference between the true slope effect size and the posterior mean of the estimated slope effect size, increased with increase in both intercept effect size and standard deviation together, except for a standard deviation value of 1. This is probably because data patterns become less clear with increase in standard deviation. Slope effect size 0-coverage was higher for the SA model than that of the SI model, and its credible intervals contained 0 more than 90% of the time. Still, both models had overcoverage of 0. As expected, the 0-coverage of slope effect sizes decreased with increase in autocorrelation, as shown in Fig. 9. However, the absolute mean relative bias of slope effect size was greater than 0.5 for all conditions. This is extremely high. The slope effect size coverage rates were all above 95% for all conditions, except the SI model only, for an autocorrelation value of 0.8. This situation seems to be exacerbated slightly by the intercept effect size. The mean relative bias of the intercept effect size (*ES*_1_) was largest for the IO model and lowest for the SI model, yet the absolute mean relative bias values were greater than the acceptable value of 0.05 for all conditions. For IO and NS, *ES*_1_ increased with an increase in true intercept effect size, but SA and SI models exhibited an opposite pattern, as shown in Fig. 9.

#### Standard deviations

The RMSE of the standard deviation (sigma) only differed to the second decimal for all cases. For the case of intercept effect size of 5, the SA model had high sigma RMSE. The mean standard deviation of *β*_11_ was affected by standard deviation and the model. As expected, this mean standard deviation increased with increase in standard deviation and model complexity. Models that estimated autocorrelations had larger mean standard deviations than those without. The same pattern was found for the mean standard deviation of *β*_21_.

### 0-Credible intervals for autocorrelation and slope

In order to better understand the behavior of the SA model with respect to 0-coverage, we investigated how many credible intervals of autocorrelation and slopes both contained the value of 0. If both autocorrelation and slopes of the second phase contained 0 in their credible interval when they should not, this indicates a possible indeterminacy problem. That is, some of the patterns in the data are sometimes interpreted as only slopes with no credit given to autocorrelation, and sometimes as only autocorrelation with no credit given to slope. The issue with this indeterminacy is that such an estimation would lead to increased type II errors. That is, concluding that there is no autocorrelation or slope when there truly is.

**Fig. 10:**
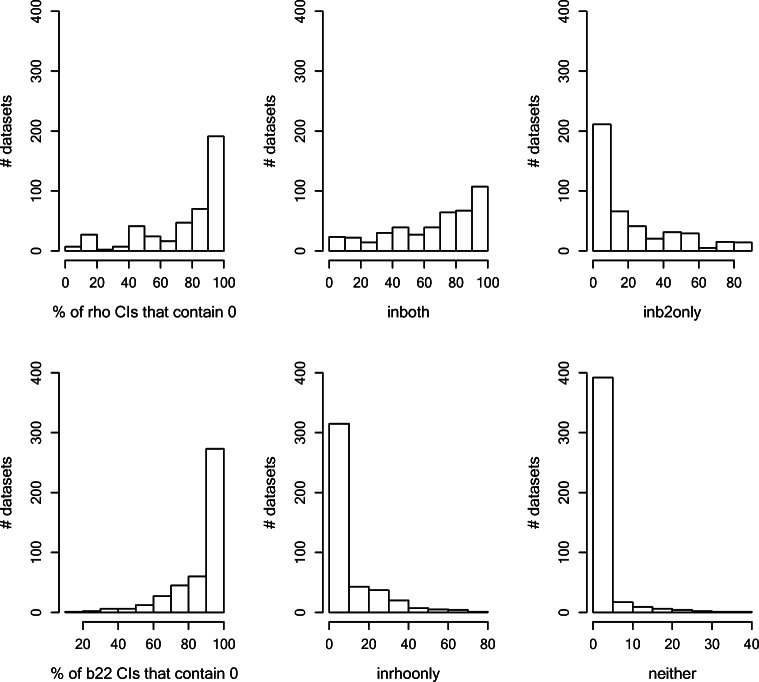
Histograms of credibility intervals (CIs) that contain 0 value in autocorrelations (rho, top left panel), second-phase slope (b22, bottom left panel), autocorrelations and second-phase slope (in both, top middle panel), in autocorrelation only (in rho-only, bottom middle panel), in second-phase slope only (in b2 only, top right panel), and in neither parameter’s (bottom right panel) CIs for the SA model

We investigated 0-coverage in datasets where neither the true autocorrelation nor the true *β*_22_ values were 0. These were 43,200 in total. Figure 10 presents the histograms for the number of datasets whose autocorrelation CIs and *β*_22_ CIs that contain 0 and the histograms for the number of datasets where both, either, or neither CIs contain 0. The histogram shows that 66.24% of the datasets’ estimates contained 0 in CIs of both parameters, 10.79% of the datasets’ estimates contained 0 in only the autocorrelation CI, and 20.9% of the datasets’ estimates contained 0 in only the *β*_22_ CI. Only 2.02% of the datasets’ estimates did not contain 0 in both autocorrelation and *β*_22_ CIs. Similarly, 66.9% of the datasets’ second-phase slope CIs contained 0 90–100% of the time. In over half of the data conditions, more than 80% of the datasets’ estimates showed that both CIs contained 0 when they should not, as shown in the histograms. This was the most prevalent case. That is, the probability of type II error for both the autocorrelation and slope of the second phase was over 0.8 in more than half of the data conditions.

Phase length (52.46%), intercept effect size (11.64%), autocorrelation (19.11%), and the interaction between phase length and autocorrelation (7.27%) explained variation in credible intervals of both the second-phase slope and autocorrelation containing 0. Data with longer phase lengths and larger true autocorrelation values had fewer cases where both credible interval estimates contained 0. Next, we considered cases where the second-phase slope credible intervals contained 0, but the autocorrelation credible intervals did not. Data with longer phase lengths and higher true autocorrelation values had more cases where the autocorrelation CIs were estimated to contain 0. Only phase length (31.72%) explained cases where the second-phase slope CIs contained 0, but the autocorrelation CIs did not. It was more common to see autocorrelation CIs contain 0 and second-phase slope not contain 0 for longer time-series and higher autocorrelations. In cases where autocorrelation CIs contained 0, but second-slope-phase CIs did not, intercept effect size (56.86%) explained most of the variance followed by the interaction of intercept effect size and autocorrelation (7.24%), and phase length and autocorrelation (6.65%). More autocorrelation CIs contained 0 when the intercept effect sizes were large.

Finally, we considered only cases where the CI estimates of both the second phase and autocorrelation did not contain 0 when they should not. Phase length (17.17%), intercept effect size (19.6%), autocorrelation (7.27%), and the interaction effects between length and autocorrelation (6.34%), length and intercept effect size (19.77%), and autocorrelation and effect size (5.33%) explained most of the variation in these estimates. Cases with longer time-series, large intercept effect sizes, and large autocorrelations had more correct CIs, that is, those where neither autocorrelation CI nor the second-phase slope CI contained 0. This shows that in general, clearer data patterns, that is, longer time-series with larger autocorrelations and intercept effect sizes, yield more power to identify slope effect size and autocorrelation.

## Conclusion

The question of which model needs to be fitted to data, in general, and SCED data, in particular, has long been a problem of interest for researchers. Often then, the question is whether we need to mimic the true model, that is, the model from which the data are generated or whether we need to find the simplest model that best explains our data. Statisticians have tended to favor the Occam’s razor approach by leaning towards selecting the simplest model, which is evident in many model fit indices such as the Akaike information criterion (AIC) and the Bayesian information criterion (BIC), which penalize models for complexity. We revisit the question posed in the title of this study as to whether we should favor accurate models or accurate estimates. This study tends towards the latter because by selecting the “correct” model, that is, the model that was used to generate the data, we obtain not only incorrect estimates but also reach incorrect decisions and potentially make type II errors. Additionally, when it comes to whether the practitioner would be concerned more with obtaining the accurate model or arriving at proper inferences and conclusions, we would always favor the latter. Thus, our recommendation is to lean toward simpler models that we can expect to yield better estimates.

The mean relative bias of intercepts and intercept effect sizes show that the intercepts only (IO) model may not be the best-suited model to estimate the parameters of a two-phase SCED model with slopes and autocorrelations. This is perhaps because there is a pattern in the datasets that is unaccounted for when using the IO model. In fact, none of the models had desirable mean relative bias for intercept effect size and slope effect size. Although the slopes and autocorrelations (SA) model had lower RMSE for autocorrelation than the no slopes but autocorrelations (NS) model, it also had substantially higher 0-coverage rates and lower coverage rates for autocorrelations with wider credible intervals and high probability of type II error rates. This indicates that the precision of the autocorrelation estimates obtained from the SA model is smaller than that of the NS model. The NS model had slightly higher second-phase intercept and intercept effect size mean relative biases than the SA model.

The slopes and intercepts but no autocorrelations (SI) model had fewer 0-coverage rates for slope effect size than the SA model, although the RMSE of the second intercept for the SA model was slightly better than that of the SI model. It also had the lowest intercept effect size mean relative bias, lower 0-coverage of slope effect size, and lowest second-phase intercept mean relative bias of all models. The main disadvantage of the SI model is that it does not estimate autocorrelations. However, practitioners are not interested in estimating autocorrelations other than to eliminate their effects when computing effect sizes for interventions. Rather, practitioners are most interested in computing and interpreting the accuracy of intercept and slope effect sizes and their credible intervals. This shows that researchers are better off choosing the slopes and intercepts model without estimating autocorrelations, rather than using a model that includes intercepts, autocorrelations, and slopes. This, of course, comes with the caveat that none of the models had desirable 0-coverage rates of slope effect sizes. The best model of the four, the SI model, still had 0-coverage rates ranging from 59% to 95%. This overcoverage of 0 value, however, was accompanied by adequate coverage of the true value. The 0-coverage has implications for false decisions about the slope effect size even when the effect size is large; however, these same credible intervals also contained the true value of the slope effect size. This implies that the credible intervals were much wider than desired and represents an avenue for further research. Perhaps more informative priors could lead to narrower credible intervals. Still, these findings only further make the case for future simulation studies to include 0-coverage rates because this diagnostic is very rarely reported in simulation studies. Therefore, we do not know how many studies that have adequate coverage rates of true values still might have undesirable 0-coverage rates.

Finally, the question is whether it is better to fit a simpler model such as the SI model even though it is not the “true” model. The advantages of fitting a simpler model to yield estimates that are more powerful outweigh the need to fit the more accurate model (SA), as our results show. Although Harrington and Velicer (2015, p. 176) noted that in single-case designs, any analysis that ignores autocorrelations is “indefensible,” Allison and Gorman (1993) suggested that failure to address and properly model trend can result in biased parameter estimates and inflated standard errors. On the other hand, Shadish, Rindskopf, and Hedges (2008) reported that modelling the trajectory of the data might reduce the inflation of autocorrelation based on model misspecification. Our results shed additional light on these viewpoints mainly because we consider credible intervals, coverage rates, and 0-coverage of CIs. We have shown that researchers may want to choose only one of these sources of trend, that is, slope in favor of autocorrelation, in order to reduce 0-coverage and reduce model complexity.

Our results also emphasize that in simulation studies, it is not adequate to observe only RMSE, standard errors, and biases as is common practice. Interval estimates and their coverage and 0-coverage rates have a more complete and sometimes even a different story to tell when evaluating the accuracy of parameters (Jennings, 1986, 1987; Natesan, 2015). Coverage rates have nominal values against which the performance of a model can be checked, unlike RMSE and biases which are unbounded statistics. We have also showed that in addition to examining coverage rates, examining 0-coverage rates is important because excessively incorrect 0-coverage rates lead to incorrectly failing to reject the null hypothesis about the parameter. Adequate coverage rates along with excessively incorrect 0-coverage rates indicate wider than necessary interval estimates.

RMSEs can only be used to compare one criterion against another to conclude which criterion had lower RMSE. Whether this low RMSE is desirable or substantially above desirable is unknown unless the value is 0. We have shown that investigating the performance of credible intervals of two variables in tandem can be helpful in evaluating model performance. In fact, in the present study, the best model in terms of RMSE (SA) is not the best in terms of coverage.

We used weakly informative priors for the study. This allows us to stay agnostic about the parameters and try to estimate them. Of course, with small sample sizes such as the ones encountered in SCEDs, researchers may find it helpful to use informative priors based on previous research. Using informative priors may yield better estimates. Again, the use of informative priors in Bayesian estimation of SCEDs needs more investigation.

The implications of our study are multi-fold: First, our study informs authors of standards such as the WWC standards that estimating slopes and autocorrelations for SCED data often yields inaccurate estimates and is not recommended. Researchers may incorrectly infer that their intervention did not have a statistically significant intervention effect as shown by the confidence intervals of the trend of the data. Second, there has been much effort spent on developing effect sizes for slopes for SCEDs. The present study indicates that any effect size that is a function of the difference between the slopes severely underestimates slope effect size by often containing 0 in its credible interval unless it ignores autocorrelations. Therefore, future studies that develop slope effect sizes for SCEDs should take 0-coverage as an important diagnostic for testing the performance of these effect sizes. Given that there is a need for statistics to be used in the domain of SCED analysis, the present study is of interest because it informs standards that should be developed that are standardized for SCED researchers to use.

## Footnotes

1 Specifying gamma distributions using mode and standard deviations is simply easier to visualize (see http://doingbayesiandataanalysis.blogspot.com/2012/08/gamma-likelihood-parameterized-by-mode.html)
